# On the Employment of Cold

**Published:** 1876-08

**Authors:** 


On the Employment of Cold.—Dr. N. S. Davis, of Chicago, in the February number of the *American Practitioner*, makes a protest against the too exclusive reliance on the thermometer in fevers, and the consequent tendency to the employment of cold as the chief therapeutic agent. His text consists of some extracts from the Toner Lecture of Dr. Horatio C. Wood, delivered in Washington, January, 1875, in which Dr. Wcod seems to regard fever and heat as almost synonymous terms. He quotes from Dr. Wood that if the head of an animal is kept for a considerable time at the temperature of 111 deg. F., the functions of the brain are suspended and the animal dies. If, however, the heat is withdrawn before death actually occurs, the animal is as well as ever the next day.

Dr. Davis claims that it is not so in fever; that in a short time after the cold applications have been withdrawn, the heat rises again. From this it is inferred that fever is something more than increased heat. He says: "We are constrained, therefore, to protest against the doctrine that 'fever and excessive bodily temperature are synonymous,' and still more against the prevalent tendency to make the mere measure of temperature the chief guide, both in diagnosis and in the application of remedies."

We cheerfully acknowledge that cold baths, large doses of quinia, digitalis, etc., technically called anti-pyrexic treatment of fever, is far more successful and safe than the system previously so much in vogue, of stuffing fever patients with egg-nog, whisky, or brandy-punch, wine and *waiting* or expectancy. But a still more safe and successful method would consist in a recognition of the fact that all general fevers result from causes acting on the properties common to all living creatures, disturbing or perverting them in such a way as to involve a simultaneous disturbance of innervation, circulation, secretion and calorification.—*St. Louis Medical and Surgical Journal.*

